# Fulminant septic shock due to *Streptococcus pneumoniae* bacteremia without initial radiographic pneumonia

**DOI:** 10.1016/j.idcr.2026.e02634

**Published:** 2026-06-13

**Authors:** Akash Kumar Sinha, Prasad Garimella

**Affiliations:** a Philadelphia College of Osteopathic Medicine - Georgia Campus, Philadelphia, PA, USA; b Northside Hospital Gwinnett, Lawrenceville, GA, USA

**Keywords:** Streptococcus pneumoniae, Septic shock, Bacteremia, Invasive pneumococcal, Disease, Radiographic pneumonia

## Abstract

*Streptococcus pneumoniae* remains a leading cause of invasive bacterial infection worldwide and is classically associated with community-acquired pneumonia. However, fulminant pneumococcal septic shock in the absence of radiographic pneumonia is uncommon and may delay diagnosis and treatment. We present the case of a 48-year-old immunocompetent female who developed severe septic shock secondary to *Streptococcus pneumoniae* bacteremia despite initially negative chest imaging for focal pneumonia. The patient presented with profound hypotension, lactic acidosis, thrombocytopenia, acute kidney injury, and acute hypoxic respiratory failure requiring vasopressor support and intensive care admission. Blood cultures grew *Streptococcus pneumoniae*. Initial chest radiography and computed tomography angiography of the chest demonstrated no focal consolidative pneumonia, although delayed bibasilar pulmonary infiltrative changes developed later during hospitalization. Extensive evaluation did not identify alternative infectious sources including endocarditis or meningitis. The patient improved rapidly with aggressive resuscitation and targeted antimicrobial therapy. This case highlights the importance of recognizing invasive pneumococcal disease even in the absence of classic radiographic findings and emphasizes that early imaging may underestimate evolving pulmonary infection in severe sepsis.

## Introduction

*Streptococcus pneumoniae* remains a major cause of invasive bacterial infections including bacteremia, meningitis, and community-acquired pneumonia despite widespread vaccination efforts [1]. Pneumococcal bacteremia is most commonly associated with radiographically apparent pulmonary infection; however, invasive pneumococcal disease may occasionally present without clear evidence of pneumonia on initial imaging studies [2]. Such presentations may delay recognition of the underlying infectious source, particularly in patients presenting primarily with septic shock physiology.

Fulminant septic shock secondary to pneumococcal bacteremia without initial radiographic evidence of pneumonia is uncommon in immunocompetent adults. Proposed mechanisms include early hematogenous dissemination preceding development of alveolar inflammatory infiltrates detectable on imaging [3]. Additionally, chest radiographs obtained early in the disease course may underestimate pulmonary involvement in severely ill patients [4].

We present a case of severe septic shock due to *Streptococcus pneumoniae* bacteremia in an immunocompetent adult patient with initially negative chest imaging for focal pneumonia who subsequently developed evolving pulmonary findings during hospitalization.

## Case presentation

A 48-year-old female with no significant past medical history presented to the emergency department with progressive weakness, sore throat, dry cough, dyspnea, and lethargy over approximately one day prior to admission. The patient had recently stopped smoking approximately one month prior to presentation after a 15-pack-year smoking history. No history of splenectomy, malignancy, chronic immunosuppressive therapy, or other known immunocompromising condition was documented. Pneumococcal vaccination status could not be definitively confirmed from the available medical record.

On arrival, the patient was critically ill with septic shock characterized by profound hypotension, tachycardia, acute hypoxic respiratory failure, severe lactic acidosis, thrombocytopenia, leukocytosis, acute kidney injury, and metabolic acidosis. Initial laboratory evaluation demonstrated lactate greater than 20 mmol/L, platelet count of approximately 46 K/µL, leukocytosis of 18.1 K/µL, elevated procalcitonin of 67.46 ng/mL, elevated INR and PT/PTT, and evidence of multiorgan hypoperfusion. Peripheral smear demonstrated marked thrombocytopenia with toxic granulation and vacuolated neutrophils concerning for severe systemic infection and early disseminated intravascular coagulation (DIC).

Physical examination demonstrated an acutely ill-appearing woman in respiratory distress. No petechiae, purpura, meningismus, focal neurologic deficits, cardiac murmurs, or skin and soft tissue infectious findings were documented. No obvious alternative infectious source was identified on examination. Respiratory viral PCR testing including SARS-CoV−2, influenza, RSV, adenovirus, and atypical respiratory pathogens was negative. Urinalysis was unremarkable.

Portable chest radiograph obtained on presentation demonstrated no focal pulmonary consolidation (Fig. 1). Computed tomography angiography of the chest similarly showed small bilateral pleural effusions with bibasilar atelectatic and mild interstitial changes but no focal lobar consolidation, cavitary lesion, or pulmonary embolism (Fig. 2).

**Fig. 1:**
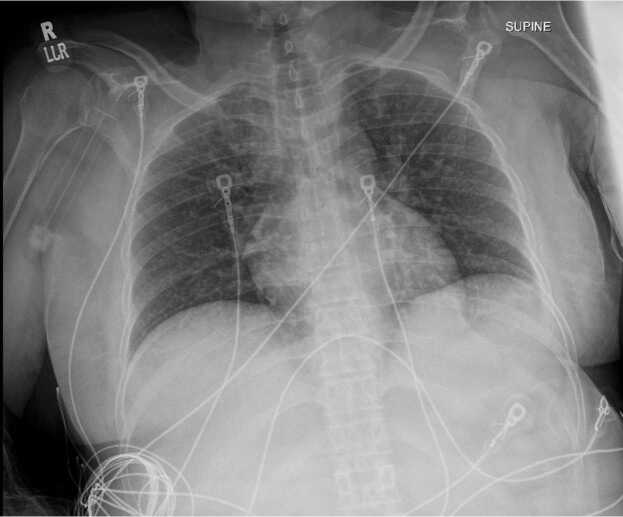
Admission chest radiograph obtained on presentation demonstrating subtle bilateral reticulonodular pulmonary changes without focal lobar consolidation in a patient with severe septic shock secondary to *Streptococcus pneumoniae* bacteremia.

**Fig. 2:**
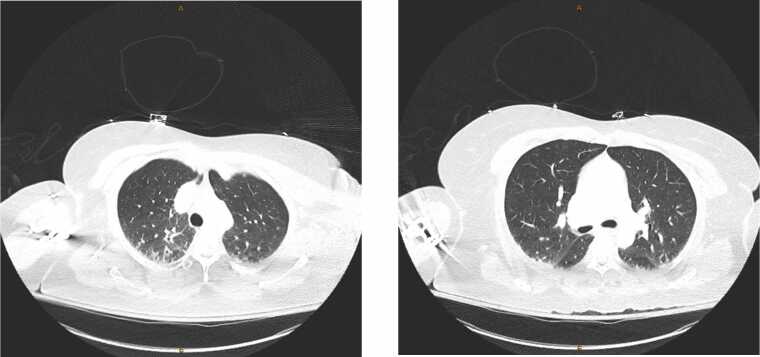
Computed tomography angiography of the chest obtained on admission demonstrating small bilateral pleural effusions and bibasilar atelectatic/interstitial changes without focal lobar consolidation or pulmonary embolism.

The patient required aggressive intravenous fluid resuscitation, broad-spectrum antimicrobial therapy with vancomycin and piperacillin-tazobactam, vasopressor support with norepinephrine, and admission to the intensive care unit. Blood cultures subsequently grew *Streptococcus pneumoniae*. Susceptibility testing demonstrated sensitivity to ceftriaxone, cefotaxime, and vancomycin with penicillin resistance. Antimicrobial therapy was narrowed to ceftriaxone 2 g intravenously daily following organism identification and susceptibility review.

Transthoracic echocardiography demonstrated preserved left ventricular ejection fraction of 55–60% without valvular vegetations or evidence of infective endocarditis. Additional evaluation did not identify meningitis, sinusitis, intra-abdominal infection, or other occult infectious foci.

During hospitalization, repeat chest imaging demonstrated evolving bibasilar infiltrative pulmonary changes consistent with delayed pulmonary involvement (Fig. 3). Repeat portable chest imaging demonstrated mild diffuse irregular bilateral lung opacities interpreted as possible evolving atypical pneumonic process versus atelectatic change. Vasopressor requirements resolved within approximately 48 h of targeted therapy and aggressive supportive care. Renal function, oxygenation, lactate levels, and thrombocytopenia progressively improved over the subsequent days. Platelet count improved from a nadir of approximately 46 K/µL to approximately 80 K/µL prior to discharge. The patient completed intravenous ceftriaxone therapy during hospitalization and was discharged home in stable condition on oral cefdinir 300 mg twice daily to complete a prolonged outpatient antibiotic course after a six-day hospitalization.

**Fig. 3:**
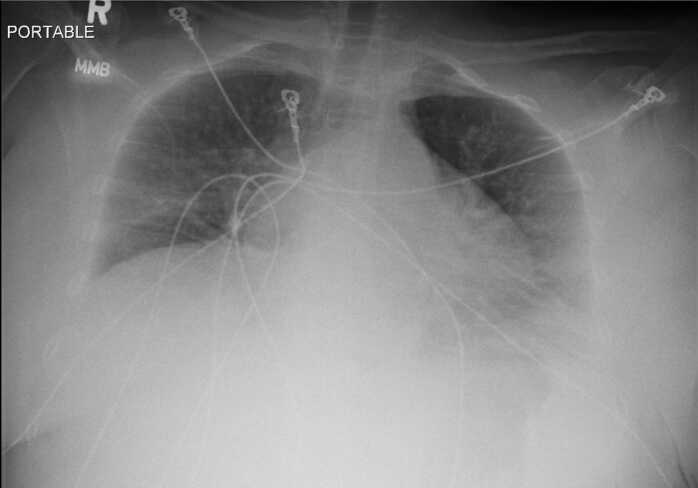
Repeat portable chest radiograph obtained later during hospitalization demonstrating evolving bilateral reticulonodular and bibasilar pulmonary opacities concerning for delayed pneumonic involvement following initially nondiagnostic chest imaging.

The overall clinical course is summarized in Table 1.

**Table 1:** Clinical timeline demonstrating progression from initial viral-like respiratory symptoms to fulminant septic shock secondary to *Streptococcus pneumoniae* bacteremia, including imaging evolution, microbiologic diagnosis, intensive care course, and clinical recovery.

**Date**	**Clinical Events**
1 day prior to admission	Viral-like respiratory symptoms with progressive weakness, cough, dyspnea, and lethargy
Admission Day	Presented in septic shock with hypotension, lactic acidosis, thrombocytopenia, acute kidney injury, and hypoxic respiratory failure
Admission Day	Chest radiograph and CTA chest without focal consolidative pneumonia
Hospital Day 1	ICU admission, norepinephrine vasopressor support, broad-spectrum antibiotics initiated
Hospital Day 1	Blood cultures positive for Streptococcus pneumoniae
Hospital Day 2	Respiratory viral panel negative; transthoracic echocardiogram without vegetations
Hospital Days 2–3	Vasopressor support discontinued with improving hemodynamics and lactate
Hospital Days 3–4	Repeat imaging demonstrated evolving bibasilar infiltrative pulmonary changes
Hospital Day 5	Platelet recovery and continued clinical improvement
Hospital Day 6	Discharged home on oral cefdinir

## Discussion

This case demonstrates fulminant septic shock secondary to invasive pneumococcal bacteremia despite initially nondiagnostic chest imaging. Although *Streptococcus pneumoniae* classically presents with radiographic evidence of community-acquired pneumonia, our patient initially demonstrated only subtle nonspecific pulmonary findings without focal lobar consolidation on chest radiography or computed tomography (Fig. 1, Fig. 2). Despite initially unrevealing imaging, she rapidly progressed to profound septic shock requiring vasopressor support and intensive care admission.

Repeat chest radiography later during hospitalization demonstrated evolving bilateral pulmonary infiltrative changes consistent with delayed pulmonary involvement (Fig. 3). Early imaging may therefore underestimate pulmonary disease during the initial phase of invasive pneumococcal infection, particularly in patients presenting early in the disease course or with overwhelming systemic inflammatory response.

Extensive evaluation did not identify an alternative infectious source. Pertinent negative findings included absence of meningismus, focal neurologic deficits, sinus tenderness, cardiac murmur, or peripheral stigmata of infective endocarditis. Transthoracic echocardiography did not demonstrate valvular vegetations. Blood cultures confirmed *Streptococcus pneumoniae*, and susceptibility testing demonstrated ceftriaxone sensitivity with penicillin resistance, allowing targeted antimicrobial therapy.

Although the patient had a history of tobacco use, no known immunocompromising condition was identified. Review of the medical history revealed no splenectomy, malignancy, chronic immunosuppressive therapy, or other recognized predisposing condition for invasive pneumococcal disease. Pneumococcal vaccination status could not be definitively confirmed from the available medical record.

The patient improved rapidly following aggressive resuscitation, vasopressor support, and antimicrobial therapy. This case highlights that invasive pneumococcal disease may present with fulminant septic shock before development of radiographically apparent pneumonia. Clinicians should maintain suspicion for invasive pneumococcal infection in patients presenting with severe sepsis and respiratory symptoms despite initially nondiagnostic chest imaging.

## Learning Points

• Pneumococcal septic shock may occur without radiographic pneumonia.
• Absence of pulmonary consolidation does not exclude invasive pneumococcal disease.
• Early recognition and prompt antimicrobial therapy are essential in septic shock.

## CRediT authorship contribution statement

**Prasad Garimella:** Supervision. **Akash Kumar Sinha:** Writing – original draft, Project administration, Methodology, Investigation, Formal analysis, Data curation, Conceptualization.

## Patient Consent

Written informed consent was obtained from the patient for publication of this case report and accompanying images. A copy of the written consent is available for review by the Editor-in-Chief of this journal on request.

## Declaration of Competing Interest

The authors declare that they have no known competing financial interests or personal relationships that could have appeared to influence the work reported in this paper.

## References

[1] Feldman C., Anderson R. (2020). Recent advances in the epidemiology and prevention of *Streptococcus pneumoniae* infections. F1000Res.

[2] Metlay J.P., Waterer G.W., Long A.C. (2019). Diagnosis and treatment of adults with community-acquired pneumonia. Am J Respir Crit Care Med.

[3] Marrie T.J., Tyrrell G.J., Garg S. (2017). Invasive pneumococcal disease in adults. Chest.

[4] Wunderink R.G., Waterer G.W. (2014). Community-acquired pneumonia. N Engl J Med.

